# Changes in Gene Expression Related to Atopic Dermatitis in Mothers and Infants Following VOC Exposure

**DOI:** 10.3390/ijms252312827

**Published:** 2024-11-28

**Authors:** Seung Hwan Kim, So Yeon Yu, Jeong Hyeop Choo, Jin Kyeong Kim, Jihyun Kim, Kangmo Ahn, Seung Yong Hwang

**Affiliations:** 1 Department of Bio-Nanotechnology, Hanyang University, Sangnok-gu, Ansan 15588, Gyeonggi-do, Republic of Korea; kandoli1@daum.net; 2 Institute of Science and Convergence Technology, Hanyang University, Sangnok-gu, Ansan 15588, Gyeonggi-do, Republic of Korea; yusso3027@naver.com; 3 Department of Molecular & Life Science, Hanyang University, Sangnok-gu, Ansan 15588, Gyeonggi-do, Republic of Korea; cnwjdguq@naver.com (J.H.C.); jink0207@naver.com (J.K.K.); 4 Department of Pediatrics, Samsung Medical Center, Sungkyunkwan University School of Medicine, Seoul 06351, Republic of Korea; jihyun77.kim@samsung.com (J.K.); kmaped@gmail.com (K.A.); 5 Department of Health Sciences and Technology, Samsung Advanced Institute for Health Sciences & Technology, Seoul 06355, Republic of Korea; 6 Department of Medicinal and Life Sciences, Hanyang University, Sangnok-gu, Ansan 15588, Gyeonggi-do, Republic of Korea; 7 Department of Applied Artificial Intelligence, Hanyang University, Sangnok-gu, Ansan 15588, Gyeonggi-do, Republic of Korea

**Keywords:** VOCs, toluene, xylene, benzene, atopic dermatitis, DNA expression, DNA methylation, gene network

## Abstract

Environmental pollutants, particularly volatile organic compounds (VOCs), are associated with various diseases, including atopic dermatitis (AD). However, despite numerous studies on AD, there is a lack of research on the impact of various environmental exposures on mothers and infants. This study, therefore, investigated the effects of maternal exposure to specific VOCs (toluene, xylene, and benzene) on the expression of AD-related genes in mothers and their infants. RNA expression levels and DNA methylation patterns were analyzed to examine the correlation between environmental exposures and AD. A multi-omics approach integrating gene expression and methylation data was additionally employed to gain a broader understanding of the genetic impact of VOC exposure. Network analysis revealed significant changes in gene expression associated with AD. For example, maternal exposure to toluene resulted in the upregulation of AQP10, which is linked to keratinocyte dysfunction, and in infants, the genes IL31RA and CCL20 were notably affected, both of which play critical roles in immune response and skin barrier function. In mothers exposed to xylene, the histamine receptor gene HRH1 was identified as a key player in influencing AD through its role in skin barrier recovery, while infants exhibited consistent network responses with upregulation of IL31RA and downregulation of TIGIT, reflecting a shared response across different xylene isomers. Interestingly, infants exposed to xylene isomers displayed nearly identical gene network patterns, suggesting developmental resistance to diverse environmental factors. No significant gene changes were identified in the benzene-exposed group. These findings suggest that exposure to specific VOCs may have different effects on gene expression related to AD, highlighting the complexity of how environmental factors contribute to disease development.

## 1. Introduction

The impact of environmental pollutants on human health has been emphasized in numerous studies, with volatile organic compounds (VOCs) emerging as a major concern [1,2]. VOCs, which include toluene, xylene, and benzene, are substances that are easily encountered in industrial activities and daily life [3,4,5,6,7]. These compounds are commonly found in both indoor and outdoor air, and their adverse effects on human health have been documented.

Exposure to VOCs can lead to various health problems with particularly pronounced effects on the immune system and skin. Toluene, xylene, and benzene are primarily absorbed into the body through the respiratory system or skin, potentially causing damage to the nervous, respiratory, and immune systems [8,9,10,11]. Pregnant women, in particular, are at higher risk, as exposure to these chemicals may adversely affect infants. Studies on the health effects of VOC exposure in both mothers and infants are, therefore, vital [12,13].

Atopic dermatitis (AD) is a chronic inflammatory skin disease that arises from the interaction between environmental and genetic factors [14,15,16]. Recent studies have highlighted the exposome framework as an effective approach for understanding the interaction between environmental exposures and genetic predispositions in AD. The exposome considers all lifetime environmental exposures, which can amplify the risk of AD [17,18,19,20,21]. Various studies have suggested that environmental pollutants contribute to the increased incidence of AD [22,23,24]. For example, VOC exposure has been associated with increased severity of AD symptoms such as chronic pruritus, itch flares, and eczematous lesions [25,26,27]. These symptoms are often linked to immune dysregulation, elevated levels of pro-inflammatory cytokines, and disruption of the skin barrier [28,29].

Epigenetic modifications, such as changes in gene expression and DNA methylation, may play crucial roles in this process. Prenatal exposure is particularly associated with alterations in the methylation patterns of various genes, which may increase susceptibility to a range of diseases [30,31]. Observing gene expression and epigenetic changes is key to understanding the biological effects of environmental pollutants. RNA expression level analysis allows the evaluation of the activity of specific genes, whereas DNA methylation analysis helps understand long-term regulatory changes in gene expression [32,33,34,35,36]. Exposure to VOCs induces gene expression and epigenetic changes that may be linked to the pathogenesis of AD.

This study aimed to investigate changes in gene expression (RNA expression) and DNA methylation levels related to AD in mothers and infants following exposure to volatile organic compounds (VOCs) such as toluene, xylene, and benzene. This study, therefore, sought to clarify the relationship between VOC exposure and the development of AD, providing foundational data for protecting the health of mothers and infants.

## 2. Results

### 2.1. Cohort Information

We conducted our study on 124 pairs of samples from the Growing Children’s Health and Evaluation of Environment (GREEN) cohort, for which comprehensive exposure information was available based on maternal data. The participant information is summarized in Table 1. Among the infants, there were 73 boys and 48 were girls, and three children whose information remained undisclosed. Most maternal participants were non-smokers, and the majority had experienced one or two pregnancies, with an average age of 33.9 years. The average exposure levels for toluene, xylene (2-MHA), xylene (3-MHA, 4-MHA), and benzene were 9.2 μg/g cr., 43.3 μg/g cr., 201.6 μg/g cr., and 0.9 μg/g cr., respectively.

### 2.2. Correlation by Exposure Substance

**Figure 1:**
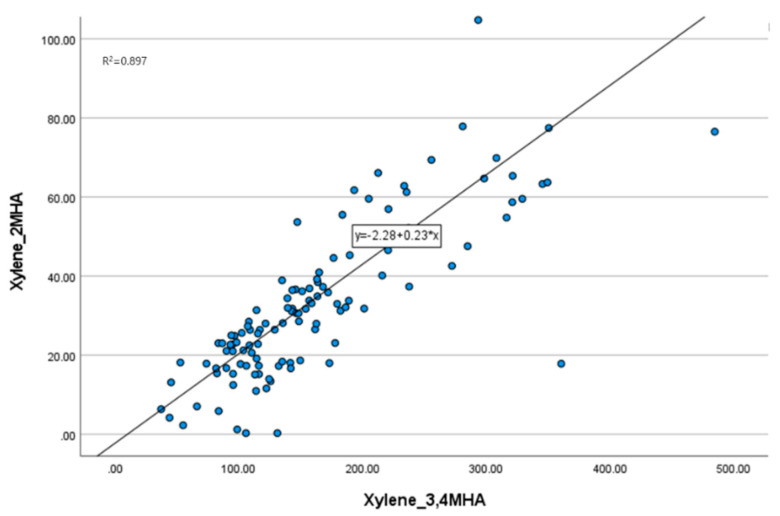
Scatter plot of two groups of xylene. The exposure levels of 2-MHA and 3,4-MHA show a positive correlation.

Before conducting the analysis, we assessed the independence of each environmental pollutant within the cohort by examining the correlation between the exposure levels. The Pearson correlation coefficients between toluene, xylene (2-MHA, 3,4-MHA), and benzene were calculated (Table 2). The correlation between toluene and benzene, excluding the metabolites 2-MHA and 3,4-MHA of xylene, was less than 0.5. In contrast, the correlation between 2-MHA and 3,4-MHA was 0.947, indicating a strong positive correlation, which was statistically significant with a *p*-value below 0.001. A scatter plot confirmed a positive correlation between the two metabolites, yielding an R^2^ value of 0.897 (Figure 1). These results demonstrate that there is no correlation between the exposure levels of substances other than xylene and its isomers. Therefore, in subsequent analyses, we did not consider the interactions between different substances.

### 2.3. Classification of Exposure Groups

Among the 124 mothers and infants with sequencing data, we classified the samples that passed quality control during the sequencing phase into high-exposure (case) and low-exposure (control) groups for the differentially expressed genes (DEG) and differentially methylated regions (DMR) analyses of VOCs (toluene, xylene, and benzene) (Table 3). The maternal case group comprised 27 individuals with an average toluene exposure level of 31.0 μg/g cr. years, with an average age of 33.8 years. The control group comprised 90 individuals with an average toluene exposure level of 2.8 μg/g cr. years, with an average age of 33.9 years. The case and control groups included 19 and 74 infants, respectively.

For xylene, specifically 2-MHA, the maternal group comprised 31 individuals with an average exposure level of 98.6 μg/g cr. years, with an average age of 34.4 years. The control group comprised 86 individuals with an average exposure level of 24.9 μg/g cr. years, with an average age of 33.6 years. In the infant group, the case group included 24 infants, while the control group included 69.

For xylene, including 2-MHA and 3-MHA, the maternal group included 31 individuals with an average exposure level of 416.9 μg/g cr. years, with an average age of 34.3 years. The control group consisted of 86 individuals with an average exposure level of 129.8 μg/g cr. years, with an average age of 33.7 years. The case group included 23 infants, whereas the control group included 70.

Finally, the maternal case group included 28 individuals with an average exposure level of 1.2 μg/g cr. and an average age of 34.2 years, whereas the control group comprised 89 individuals with an average exposure level of 0.8 μg/g cr. years, with an average age of 33.7 years. In the infant group, the case group included 20 infants, while the control group included 73.

### 2.4. DEG Analysis

The VOC exposure groups were classified into high- and low-exposure groups for DEG analysis (Table 4). A cutoff of |fold change (FC)| < 1.5 and a *p*-value < 0.05 were established for the DEG analysis. In the maternal group, the numbers of upregulated genes were 326 for toluene, 354 for xylene (2-MHA), 388 for xylene (3-MHA, 4-MHA), and 229 for benzene, whereas the numbers of downregulated genes were 163, 177, 95, and 244, respectively.

In the infant group, the numbers of upregulated genes were 163 for toluene, 166 for xylene (2-MHA), 148 for xylene (3-MHA and 4-MHA), and 313 for benzene, with downregulated gene counts of 278, 401, 473, and 191, respectively.

In mothers, the number of upregulated genes was higher for toluene and the two xylene metabolites, whereas the number of downregulated genes was higher for benzene. In contrast, there were more downregulated genes for toluene and the two xylene metabolites in infants, whereas the number of upregulated genes was higher for benzene.

### 2.5. DMR Analysis

DMR analysis was conducted by classifying the samples into high- and low-exposure groups following the same criteria as in the DEG analysis (Table 5). A cutoff of |FC| < 2 and a *p*-value < 0.05 were established for the DMR analysis. The number of hypermethylated regions in the maternal group was 14,391 for toluene, 4178 for xylene (2-MHA), 15,772 for xylene (3,4-MHA), and 2480 for benzene, whereas the number of hypomethylated regions was 8512, 10,803, 10,479, and 15,059, respectively.

In the infant group, the number of hypermethylated regions was 1864 for toluene, 2819 for xylene (2-MHA), 2923 for xylene (3,4-MHA), and 17,784 for benzene, with 18,948, 16,385, 16,591, and 2279 hypomethylated regions, respectively.

### 2.6. Integrated and Functional Analysis

An integrated analysis was performed using the gene lists derived from the DEG and DMR analyses. Common genes between the upregulated and hypomethylated regions, as well as those between the downregulated genes and hypermethylated regions were identified (Table 6). The number of downregulated genes exceeded that of the upregulated genes in the infant benzene exposure group, whereas there were more upregulated genes than downregulated genes in the other groups.

**Figure 2:**
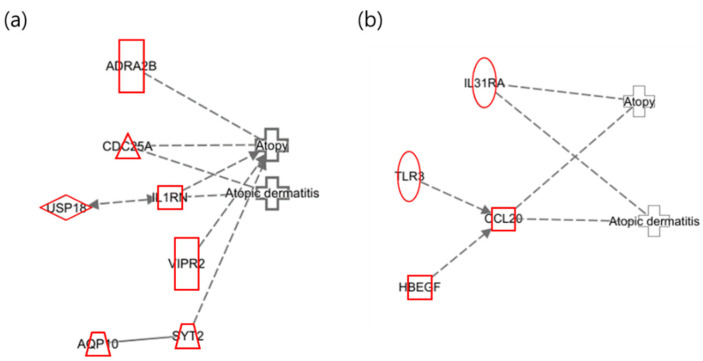
Network of toluene exposure related to “Atopy” and “Atopic Dermatitis”. (**a**) Maternal network for atopy and atopic dermatitis in relation to toluene exposure; (**b**) infant network for atopy and atopic dermatitis in relation to toluene exposure.

The identified genes were analyzed using IPA (ver. 17.6) with the diseases set to “Atopy” and “Atopic Dermatitis”, resulting in a detailed network (Figure 2). The areas highlighted in red in the network represent the upregulated genes.

In the maternal network of toluene exposure, as presented in Figure 2a, AQP10 was confirmed to be upregulated, which has been reported to exacerbate psoriasis-like acanthosis by influencing keratinocyte hyperproliferation [37]. IL31RA and CCL20 were found to be upregulated in the infant network (Figure 2b). IL31RA is a part of the JAK-STAT signaling pathway, which can cause skin barrier dysfunction [38]. CCL20 is additionally involved in the formation and function of lymphoid tissue and is upregulated in patients with atopy [39].

**Figure 3:**
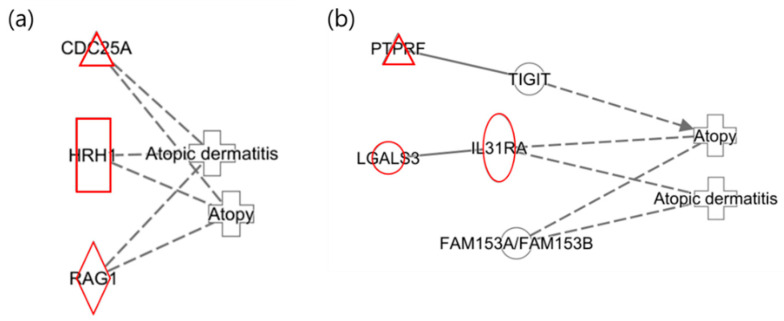
Network analysis of exposure to xylene (2-MHA) related to “Atopy” and “Atopic Dermatitis”. (**a**) The maternal network in response to xylene (2-MHA) exposure regarding atopy and atopic dermatitis. (**b**) The infant network in response to xylene (2-MHA) exposure concerning atopy and atopic dermatitis.

The following illustrates the networks of the maternal and infant responses to 2-MHA, a metabolite of xylene. Figure 3a depicts the maternal network in relation to xylene (2-MHA) exposure, where the gene HRH1 was identified as a histamine receptor that acts as an antagonist in the recovery of the skin barrier, according to previous studies [40]. This finding is consistent with the results of the network analysis. Figure 3b presents the infant network in response to xylene (2-MHA) exposure, highlighting that TIGIT plays a role in inhibiting CD4 + T-cell responses and is downregulated in psoriasis, corroborating findings related to AD [41]. As previously mentioned, IL31RA is included in the JAK-STAT signaling pathway and was upregulated in this network analysis [38].

**Figure 4:**
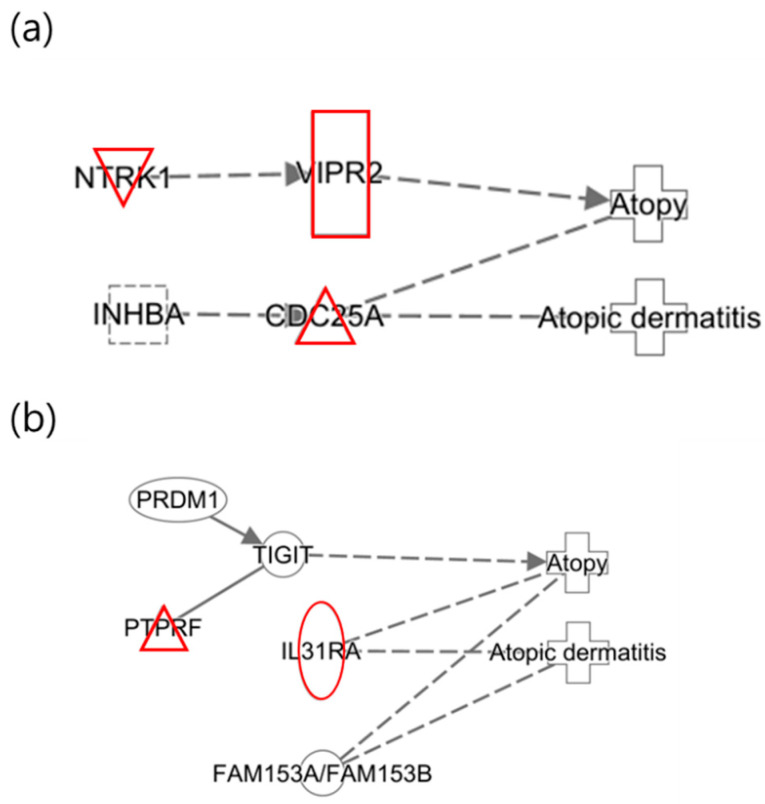
Network analysis for exposure to xylene (3-MHA, 4-MHA) related to “Atopy” and “Atopic Dermatitis”. (**a**) Network for the mother and (**b**) network for the infant, where the red color indicates upregulated genes.

The following illustrates the network analysis for exposure to 3-MHA and 4-MHA, which are metabolites of xylene, in both maternal and infant samples. Figure 4a depicts the maternal network, in which NTRK1 is not directly connected to the atopy node but is involved in cell differentiation. Previous studies have indicated that NTRK1 is upregulated in allergic inflammatory tissues [42]. In Figure 4b, the infant network reveals the downregulation of TIGIT, which inhibits CD4 T cell responses and has also been shown to be downregulated in psoriasis, according to prior research [41]. Additionally, IL31RA was upregulated, confirming that the results for these two nodes are consistent with previous findings [38].

**Figure 5:**
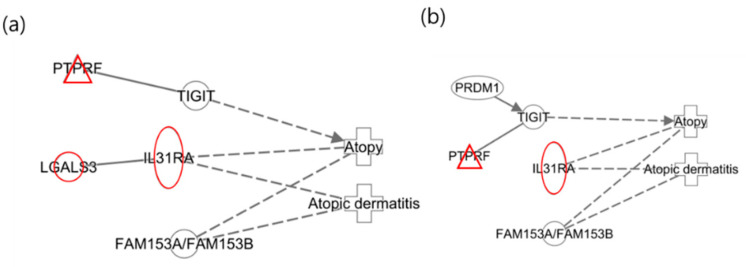
Network analysis for xylene isomers in infants. (**a**) Network for 2-MHA exposure; (**b**) net-work for 3-MHA and 4-MHA exposures.

An additional interesting finding from the study of xylene isomers was that the networks of the infants were nearly identical (Figure 5). In contrast to the different networks observed in mothers, similar networks were identified in two distinct groups of infants. This suggests that infants who have not yet been exposed to a wide range of environmental factors may experience effects comparable to those of xylene isomers, considering they are less influenced by other substances.

**Figure 6:**
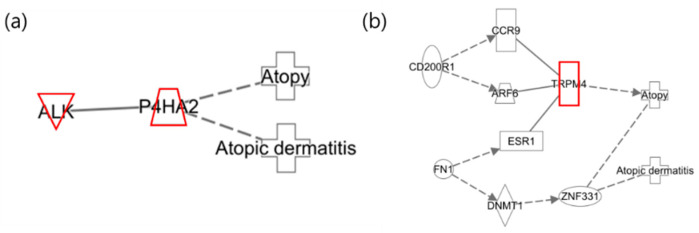
Network analysis of benzene exposure related to “Atopy” and “Atopic Dermatitis”. (**a**) Network for mothers and (**b**) network for infants, where red indicates upregulated genes.

The following illustrates the network of benzene exposure in mothers and infants. Figure 6a shows the network of mothers where no relevant associations with AD were identified. In Figure 6b, which depicts the infant network, CD200R1, FN1, TRPM4, and ZNF331 are highlighted as the results of the integrative analysis, while the remaining nodes were included to represent the network. Among the nodes identified in the integrative analysis, CD200R1 and FN1 were found to be downregulated. Previous studies have demonstrated that CD200R1 regulates immune responses and is upregulated in AD phenotypes, whereas FN1 is involved in cell adhesion, growth, migration, and differentiation and is also upregulated in AD responses. These findings are, therefore, inconsistent with the results of the current integrative analysis [43,44].

## 3. Discussion

Research has extensively explored the potential onset of environment-induced diseases due to exposure to environmental pollutants. AD is particularly prevalent, affecting approximately 20% of infants and 10% of adults [45]. Despite numerous studies on AD, there is a lack of research on the impact of various environmental exposures on mothers and infants. This study, therefore, aimed to investigate the effects of environmental pollutants on AD in mothers and their infants. This study focused on the exposure of mothers to volatile organic compounds (VOCs), specifically toluene, xylene, and benzene, by measuring RNA expression levels and DNA methylation in both mothers and infants. We integrated these data to examine their correlations with atopy. This approach aims to verify changes in gene expression more accurately through multi-omics analysis than through single-omics [46,47]. By comparing the genes identified in this analysis with those identified in previous studies, we aimed to predict the correlation between environmental pollutant exposure and AD.

We examined the association between AD and exposure to toluene, xylene, and benzene. In this analysis, we measured the urinary concentrations of the three isomers of the xylene metabolites, 2-MHA, 3-MHA, and 4-MHA, together with the concentrations of 3-MHA and 4-MHA.

In terms of maternal exposure to toluene, we observed that AQP10 was upregulated in mothers, which has been shown to exacerbate psoriasis-like ichthyosis by affecting keratinocytes [25]. In infants, we found that IL31RA and CCL20 had similar effects on AD, which is consistent with previous findings [38,39]. Notably, CCL20 plays a crucial role in the formation and function of the mucosal lymphoid tissue [39].

In the network of mothers exposed to xylene (2-MHA), HRH1 was identified as a histamine receptor, which has been found to potentially influence AD by acting as an antagonist in barrier recovery [40]. The results for infants confirmed that TIGIT was downregulated, and IL31RA was upregulated, which is consistent with previous findings. TIGIT plays a role in inhibiting CD4 + T cell responses and has been reported to be downregulated in psoriasis [41]. Similarly, IL31RA, as previously described, is part of the JAK-STAT signaling pathway and can negatively affect skin barrier function, contributing to the exacerbation of AD [38].

Upregulation of NTRK1 was confirmed in the group exposed to xylene (3-MHA and 4-MHA). NTRK1 is involved in cell differentiation and has been reported to be upregulated in allergic inflammatory tissues [42], which is consistent with the current findings. In infants, both IL31RA and TIGIT were identified as being upregulated and downregulated, respectively, corroborating the results of previous research [38,41].

No genes associated with AD were identified in the benzene-exposed group in either the maternal or infant results.

However, these results do not necessarily imply that exposure to these substances directly influences the development of AD. We conducted differential expression gene (DEG) and differential methylation region (DMR) analyses in this study, followed by an integrated analysis of the results. Nonetheless, even with the integration of these two omics approaches, there are limitations in capturing all the genetic and biochemical changes occurring in vivo. Therefore, the effects of environmental pollutants on AD may be manifested through different biochemical pathways that were not fully elucidated in this analysis. The impact of environmental pollutants on human health is complex, with numerous factors influencing disease development. However, some of these factors are not well understood, highlighting the need for further research.

Our research suggests that maternal exposure to environmental pollutants, particularly VOCs, could influence the gene expression and immune responses associated with AD in both mothers and their infants. While this study primarily focused on the early stages of AD development, the observed gene alterations in both maternal and infant samples may not only contribute to the initiation of AD but could also affect the progression of the disease. For example, the upregulation of IL31RA and downregulation of TIGIT in infants suggests a potential link to the exacerbation of AD symptoms and could be involved in the chronic progression of the disease. Furthermore, understanding these molecular changes may lead to the identification of biomarkers for early detection and the development of targeted therapeutic strategies for AD, especially in high-risk populations exposed to environmental pollutants.

## 4. Materials and Methods

### 4.1. Human-Derived Samples

This study was conducted on mothers and infants enrolled in the GREEN cohort (IRB #2016-12-111) at Samsung Medical Center (SMC) from 2017 to 2021. Whole blood and urine samples were collected from 124 mothers, and umbilical cord blood samples were obtained from 124 infants. Blood samples were collected in heparin tubes (BD, Franklin Lakes, NJ, USA) and stored at 4 °C until PBMC isolation, which was performed within 24 h.

The concentrations of toluene, xylene, and benzene in the urine samples were measured by liquid chromatography–mass spectrometry (LC–MS). For toluene and benzene, the urinary concentrations of their metabolites, toluene metabolite (BMA) and benzene metabolite (PMA), were measured and adjusted for creatinine levels. The concentrations of metabolites of the three isomers were assessed for xylene. The urinary concentrations of 2-MHA, 3-MHA, and 4-MHA were measured together with those of 3-MHA and 4-MHA. Xylene metabolites, similar to other substances, were also creatinine-adjusted.

### 4.2. Assessment of Exposure Independence by Substance

To assess the independence of exposure to toluene, xylene, and benzene, Spearman’s correlation coefficients were calculated using SPSS (ver. 27) to evaluate the correlation between the levels of exposure to each substance.

### 4.3. The Classification of Exposure Groups

The degree of exposure to environmental pollutants was assessed based on maternal exposure levels. We measured the exposure levels in human-derived samples to investigate the association between exposure to toluene, xylene, and benzene and AD. Participants were classified into low-exposure and high-exposure groups according to their exposure levels, which were quantified as follows: PMA: 6.139 µg/g creatinine, 2-MHA: 48.979 µg/g cr., 3,4-MHA: 217.413 µg/g cr., and BMA: 1.173 µg/g cr.

### 4.4. RNA-Seq (RNA Preparation, Library Construction, Sequencing)

Total RNA was extracted from peripheral blood mononuclear cells (PBMCs) isolated from whole blood using the TRIzol reagent (Invitrogen, Carlsbad, CA, USA). The concentration of the extracted RNA was measured using a Nanodrop spectrophotometer (Thermo Fisher Scientific, Waltham, MA, USA), and only samples with an RIN value of 7 or higher, as determined using a 2100 Bioanalyzer (Agilent, Santa Clara, CA, USA), were used for subsequent analyses.

RNA libraries were prepared using a TruSeq Stranded mRNA Library Preparation Kit (Illumina, San Diego, CA, USA) according to the manufacturer’s instructions. A total of 50 μL of RNA with a concentration of 600 ng/μL or higher was used for fragmentation, first-strand cDNA synthesis, second-strand cDNA synthesis, 3′ end adenylation, adapter ligation, and enrichment. The resulting libraries were sequenced on a NovaSeq 6000 platform (Illumina) using paired-end sequencing with a read length of 101 bp.

### 4.5. DEG Analysis

DEG analysis was performed using the FASTQ files obtained from the sequencing output. The quality of raw data was assessed using FastQC (ver. 0.11.9), and adapter sequences were removed using the Trimmomatic software (Ver. 0.40). The reference adapter sequence used for Trimmomatic was TruSeq3-PE. fa, which was included in the program. Before the alignment step, an index was generated using STAR (ver. 2.7.8a). The generated index and reference sequences (hg38) were aligned. The “ReadsPerGene.out.tab” files produced for each sample were consolidated for subsequent DEG analysis. The DEG analysis was conducted using EdgeR (ver. 3.32.1) for R software (ver. 4.3.1), with a cutoff of *p*-value < 0.05 and fold change (FC) > 1.5.

### 4.6. MeDIP-Seq (DNA Preparation, Library Construction, Sequencing)

Peripheral blood mononuclear cells (PBMCs) were isolated from whole blood collected in heparin tubes, and genomic DNA (gDNA) was extracted. The gDNA was isolated from PBMCs using the Invitrogen PureLink Genomic DNA Mini Kit (Thermo Fisher Scientific). The purity of the gDNA was assessed using a Nanodrop (Thermo Fisher Scientific), and its concentration was measured using a dsDNA HS Assay Kit (Thermo Fisher Scientific) on a Qubit (Thermo Fisher Scientific).

Genomic DNA (gDNA) was prepared for MeDIP-seq library construction. The library was created using a TruSeq ChIP Library Preparation Kit (Illumina, San Diego, CA, USA) following the manufacturer’s instructions. A total of 2 µL of gDNA was mixed with 198 µL of working solution and subjected to fragmentation using a Bioruptor (Diagenode, Seraing, Belgium) under the conditions of 30 s on and 30 s off for 30 cycles, targeting a concentration of 500 ng/µL or higher. The fragmented DNA was subjected to immunoprecipitation using an anti-5-mC antibody. Subsequently, adaptor- and index-ligated libraries were constructed using TruSeq RNA UD Indices (Illumina). The resulting library was amplified by PCR with a size selection step to isolate fragments within the range of 300–350 bp to minimize potential sequencing bias. Size selection was performed using the Pippin Prep (Sage Science, Beverly, MA, USA). The final library was sequenced on a NextSeq 500 platform (Illumina) using paired-end sequencing with a read length of 2 × 75 bp.

### 4.7. DMR Analysis

The FASTQ files generated by sequencing were assessed for raw data quality using FastQC (Ver. 0.11.9). Subsequently, Trimmomatic (Ver. 0.40) was used to remove adapter sequences and low-quality score sequences from the raw data. The reference adapter sequence used was TruSeq3-PE.fa, which is included in the Trimmomatic program. Subsequently, an index was generated using the Bowtie 2 software (Ver. 2.4.2) with the hg38 reference sequence, and alignment was performed. The resulting SAM files were converted to BAM files using SAMtools (Ver. 1.11.0), and duplicate sequences were removed using Picard (ver. 2.25.0).

DMR analysis of the MeDIP-seq data was conducted using the MEDIPS package (ver. 1.42.0) for R software (Ver. 4.3.1). The analysis was performed with a window size of 100 bp, applying a cutoff of *p*-value < 0.05 and a fold change (FC) > 2. In the DMR analysis conducted on maternal samples, only autosomes and the X chromosome were analyzed, whereas the analysis of infant DMRs included autosomes as well as the X and Y chromosomes.

### 4.8. Integrated and Functional Analysis

We matched the genes identified in the DMR analysis with those from the DEG analysis. Specifically, upregulated genes from the DEG analysis were paired with hypomethylated genes from the DMR analysis, while downregulated genes from the DEG analysis were paired with hypermethylated genes from the DMR analysis. Subsequently, we input the resulting gene lists into Ingenuity Pathway Analysis (IPA) software (Ver. 17.6) to analyze networks and pathways associated with AD.

## 5. Conclusions

This study used gene network analysis to investigate the correlation between maternal exposure to volatile organic compounds (VOCs), including toluene, xylene, and benzene, and the development of atopic dermatitis (AD) in both mothers and infants. The results revealed a significant association between maternal exposure to toluene and xylene and the occurrence of AD in both mothers and infants. Specifically, the upregulation of genes such as AQP10 in mothers and IL31RA and CCL20 in infants was observed, suggesting a potential role of these genes in the development and exacerbation of AD. In contrast, benzene exposure did not show a significant correlation with AD in either group. These findings suggest that specific VOCs, such as toluene and xylene, may have distinct effects on AD risk, pointing to the importance of addressing environmental pollutants in AD prevention strategies. Furthermore, the study emphasizes the need for further investigation into the molecular mechanisms and biochemical pathways influenced by VOC exposure, which could provide deeper insights into the pathophysiology of AD and pave the way for the development of more effective, targeted interventions for those at high risk.

## Figures and Tables

**Table 1:** Information on participants in the GREEN cohort.

Cohort Information *n* = 124	Number or Mean ± SD
**Infant**	
Unknown	3 (2.4%)
Boys	73 (58.9%)
Girls	48 (38.7%)
**Maternal**	
**Smoking**	
No	114 (91.9%)
Yes	10 (8.1%)
**Childbirth**	
1	65 (52.4%)
2	42 (33.9%)
3	12 (9.7%)
4	4 (3.2%)
5	1 (0.8%)
**Age**	33.9 ± 3

**Table 2:** Correlation analysis between exposure substances.

		Toluene	Xylene 2-MHA	Xylene 3,4-MHA	Benzene
**Toluene**	Pearson	1			
	*p*-value				
**Xylene** **2-MHA**	Pearson	0.165	1		
	*p*-value	0.066			
**Xylene** **3,4-MHA**	Pearson	0.189	0.947	1	
	*p*-value	0.036	0		
**Benzene**	Pearson	0.008	0.258	0.194	1
	*p*-value	0.928	0.004	0.031	

**Table 3:** Classification of exposure groups for VOCs.

VOCs	High	Low
**Maternal**		
**Toluene (*n*)**	27	90
**Toluene (μg/g cr.)**	31.0 ± 92.3	2.8 ± 1.4
**Age**	33.8 ± 3.1	33.9 ± 3.4
**Xylene (*n*)** **2-MHA**	31	86
**Xylene (μg/g cr.)**	98.6 ± 90.6	24.9 ± 11.3
**Age**	34.4 ± 3.8	33.6 ± 3.2
**Xylene (*n*)** **3,4-MHA**	31	86
**Xylene (μg/g cr.)**	416.9 ± 394.2	129.8 ± 40.6
**Age**	34.3 ± 3.9	33.7 ± 3.1
**Benzene (*n*)**	28	89
**Benzene (μg/g cr.)**	1.2 ± 1.4	0.8 ± 0.7
**Age**	34.2 ± 3.9	33.7 ± 3.2
**Infant**		
**Toluene (*n*)**	19	74
**Xylene (*n*)** **2-MHA**	24	69
**Xylene (*n*)** **(3,4-MHA)**	23	70
**Benzene (*n*)**	20	73

**Table 4:** DEG in mothers and infants in relation to VOC exposure groups.

VOCs	Total DEG	Up Regulated	Down Regulated
**Maternal**			
**Toluene**	489	326	163
**Xylene** **(2-MHA)**	531	354	177
**Xylene** **(3,4-MHA)**	483	388	95
**Benzene**	473	229	244
**Infant**			
**Toluene**	441	163	278
**Xylene** **(2-MHA)**	567	166	401
**Xylene** **(3,4-MHA)**	621	148	473
**Benzene**	504	313	191

**Table 5:** DMR in mothers and infants in relation to VOC exposure groups.

VOCs	Hyper Methylated	Hypo Methylated
**Maternal**		
**Toluene**	14,391	8512
**Xylene** **(2-MHA)**	4178	10,803
**Xylene** **(3,4-MHA)**	15,772	10,479
**Benzene**	2480	15,059
**Infant**		
**Toluene**	1864	18,948
**Xylene** **(2-MHA)**	2819	16,385
**Xylene** **(3,4-MHA)**	2923	16,591
**Benzene**	17,784	2279

**Table 6:** Number of genes from integrated analysis of DEG and DMR for VOCs.

VOCs	Upregulation and Hypomethylated	Downregulation and Hypermethylated
**Maternal**		
**Toluene**	81	74
**Xylene** **(2-MHA)**	110	25
**Xylene** **(3,4-MHA)**	139	38
**Benzene**	66	25
**Infant**		
**Toluene**	86	17
**Xylene** **(2-MHA)**	71	44
**Xylene** **(3,4-MHA)**	52	48
**Benzene**	34	81

## Data Availability

The data presented in this study are available upon request from the corresponding author.
